# Positive psychological well-being predicts lower severe pain in the general population: a 2-year follow-up study of the SwePain cohort

**DOI:** 10.1186/s12991-019-0231-9

**Published:** 2019-05-31

**Authors:** Britt Larsson, Elena Dragioti, Björn Gerdle, Jonas Björk

**Affiliations:** 1 Pain and Rehabilitation Centre, and Department of Medical and Health Sciences, Linköping University, Linköping, Sweden; 2 Division of Occupational and Environmental Medicine, Lund University, Lund, Sweden; 3 Clinical Studies Sweden, Forum South, Skåne University Hospital Lund, Lund, Sweden

**Keywords:** Chronic pain, Positive well-being, Positive outcomes, Cohort, Pain severity, Multimorbidity

## Abstract

**Background:**

Positive psychology indicators like well-being and life satisfaction may play a pivotal role in pain-related outcomes. In this study, we aimed to examine the prospective associations of positive well-being and life satisfaction with pain severity.

**Methods and Subjects:**

This longitudinal study, with a follow-up of 2 years, included 9361 participants (4266 males, 5095 females; mean age: 52.5 years; SD: 17.5) without and with chronic pain (CP) at baseline. All analyses were stratified by the two sub-cohorts—participants without CP (sub-cohort 1) and participants with CP (sub-cohort 2) at baseline. The predictive associations, assessed using ordinal regression in a Generalized Linear Model, were adjusted for baseline potential confounders and reported as odds ratios (ORs) with corresponding 95% confidence intervals (CIs).

**Results:**

After adjustments, in sub-cohort 1 positive well-being at baseline was associated with lower severe pain at follow-up compared to participants with severe distress (OR: 0.64; 95% CI 0.49–0.84; *p* < 0.001). In sub-cohort 2, both positive well-being and life satisfaction at baseline were associated with lower severe pain at follow-up compared to participants with severe distress and not satisfied with life (OR: 0.80; 95% CI 0.65–0.98; *p* = 0.031 and OR: 0.82; 95% CI 0.69–0.96; *p* = 0.014, respectively).

**Conclusions:**

Positive well-being is predictive of lower pain severity both among participants without and with CP at baseline, whereas life satisfaction was found predictive of lower pain severity only for subjects with CP. Future research should emphasize implementing treatments associated with promoting and maintaining positive well-being and life satisfaction in patients who suffer from chronic pain and in risk populations.

**Electronic supplementary material:**

The online version of this article (10.1186/s12991-019-0231-9) contains supplementary material, which is available to authorized users.

## Background

Psychological well-being (hereafter referred to as well-being) can be described as having positive emotions (e.g. contentment and happiness), positive life evaluations (e.g. life satisfaction), and functioning well in daily life [1, 2]. This description falls within the hedonic perspective and essentially differs from two other perspectives of well-being—the eudaimonic, a perspective that focuses on purpose in life/optimism, and the social, a perspective that focuses on good social integration/contribution [1, 2]. In any case, well-being is referred to the presence of positive emotions and evaluations and not just absence of negative ones such as anxiety and depression [3]. Well-being is grounded in positive psychology, a scientific discipline that promotes qualities such as positive feelings, perceptions, and behaviours required for people to flourish [4].

Today, well-being has become a critical issue in positive health outcomes [1, 3]. Well-being is considered to be a key factor connected with better health outcomes such as strong health, resilience, recovery, and longevity in both healthy and diseased populations [1, 3, 5, 6]. Such outcomes are of major importance to professionals, patients, and health systems because the personal and economic burden related to ill-being is massive [1]. Cross-sectional and longitudinal studies have demonstrated positive associations between well-being and immune function and neuroendocrine regulation [7, 8], better health behaviours and sleep [7, 9, 10], lower levels of infection [7, 8], decreased risk of illness [7, 11–13], less morbidity and lower mortality [5, 6], lower disability [14, 15], decreased distress [16], and increased longevity [17]. Meta-analyses also support the protective role of well-being with respect to overall as well as cardiovascular mortality [18], survival [3], personal failures across life span [19], cancer outcomes, negative pregnancy outcomes, and physical symptoms [20].

Given this background, well-being is likely to be important to monitor when considering possible interventions in pain populations with high disability and health care costs [21, 22]. However, there is only limited evidence that suggests that well-being may have a crucial protective role in pain-related outcomes. A cross-sectional study suggested that positive well-being is associated with low pain intensity and depression [23]. Another cross-sectional study highlighted the importance of the association between life satisfaction and low pain intensity and depressive symptoms [24]. A feasibility trial investigating a tailored positive psychology intervention also resulted in lower pain intensity and better pain control in patients with chronic pain compared to controls [25]. Two other prospective studies provided evidence for an association between positive well-being and reduced risk of incident arthritis and arthritis disability [13, 14].

Uncertainty also exists with respect to the associations among diseased populations, whereas the associations among healthy populations are quite firm [1]. Additionally, predictive associations of well-being with pain-related outcomes are quite sparse. Therefore, this study focuses on the following two aims: (1) to investigate the baseline (T0) association of positive well-being and life satisfaction with five grades of pain severity at a 2-year follow-up (T1) in a large cohort of the general population without chronic pain at *T*0 (sub-cohort 1) and with chronic pain at T0 (sub-cohort 2), and (2) to assess these associations with respect to changes in pain severity at the 2-year follow-up. We hypothesized that positive well-being and life satisfaction are associated with decreased risk (i.e. protective factors) of severe pain in both sub-cohorts at T1 after controlling for baseline confounders.

## Materials and methods

### Participants and procedures

Based on a large Swedish population-based cohort study, [26, 27] this study assesses biopsychosocial aspects of pain in a sampling frame gathered from the Swedish Total Population Register. No longitudinal analyses have been reported for this cohort. Baseline data (T0) were collected using a representative stratified random sample of 34,000 individuals from the general population in south-eastern Sweden. The random sampling was stratified by gender and municipality to reach individuals living in urban and rural areas [26]. Data were collected by Statistics Sweden. The selected individuals received a postal questionnaire in March 2013, which could be returned either by post or electronically. A reminder was sent to non-responders after 2 weeks; if necessary, another reminder was sent 2 weeks later. The collection of questionnaires ended in May 2013. Follow-up data (T1) were collected 2 years later. Only individuals who completed and returned the first questionnaire were eligible to participate in the follow-up assessment. Eligible individuals received a postal survey in March 2015, which could be returned by post or electronically. Two reminders were sent. The collection of the follow-up data ended in May 2015. Both surveys at T0 and T1 included the same questions. The survey for this study is described below. Completion of the postal survey was deemed to be the agreement of participant’s informed consent. The study was approved by the local ethics committee of Linköping University, Sweden (Dnr: 2011 72/31), and this research was conducted in accordance with the Declaration of the World Medical Association.

**Fig. 1:**
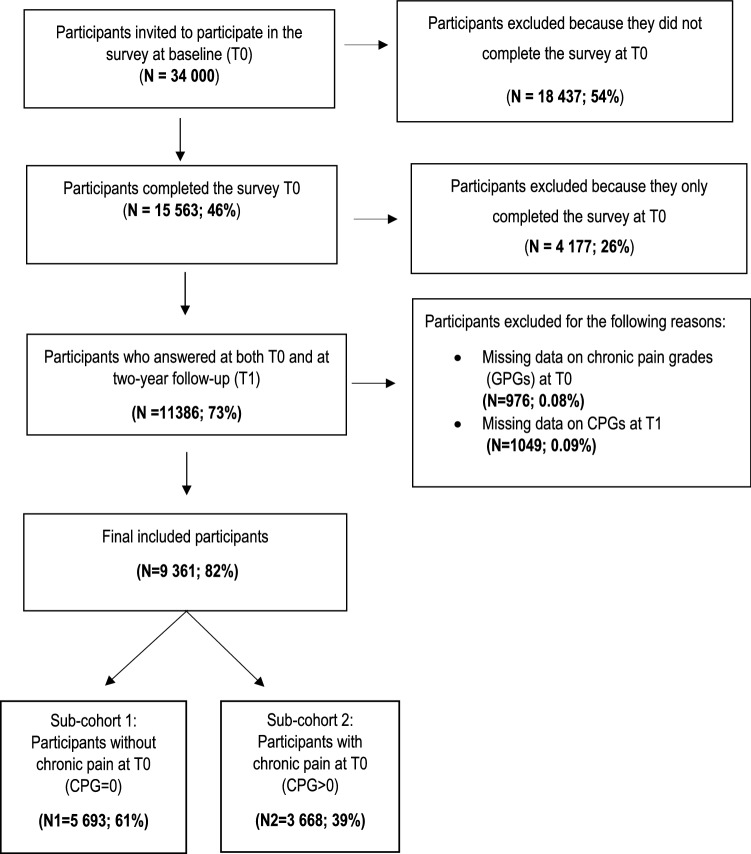
Flow chart outline of the inclusion of participants for this study. *CPGs* Chronic pain grades, T0 baseline, T1 2-year follow-up

For the present longitudinal study, inclusion criteria were age between 18 and 85 years and individuals who answered at both T0 and T1 regardless of their pain status (Fig. 1). Chronic pain (CP) was defined by a single question regarding the presence and duration of pain: “Do you usually have pain?” Three response options were available: (1) no; (2) yes, with less duration than 3 months; and (3) yes, with duration of more than 3 months. Individuals who answered 3 were classified as CP, and individuals who answered 1 or 2 were classified as no CP. Hence, this study had two sub-cohorts: sub-cohort 1—individuals without CP at baseline irrespective of their CP status at follow-up; and sub-cohort 2—individuals with CP at baseline irrespective of their CP status at follow-up (Fig. 1). This strategy allowed us to include those individuals who shift from no pain at T0 to CP at T1 and vice versa and to examine whether the associations for these two sub-cohorts differ.

Reporting of the results from this study was done in accordance with the Strengthening the Reporting of Observational Studies in Epidemiology (STROBE) statement (see Additional file 1) [28].

## Measurements

### Outcome variable

#### Pain severity

The Swedish version of the chronic pain grade (CPG) scale was chosen to capture pain severity at T0 and T1. CPG was designed to assess global pain severity in three dimensions: intensity, persistence, and disability [29–31]. Previous reports indicated that CPG is a valid and reliable instrument [32–35].

The intensity dimension (denoted CPG intensity) was composed of three items: (1) average pain intensity for the previous 7 days using a numeric rating scale (NRS; 0 = no pain and 10 = worst imaginable pain); (2) pain intensity at its worst the previous 4 weeks using the NRS; and (3) average pain intensity previous 4 weeks using the NRS. CPG intensity was calculated as the average of these three scales and multiplying this by ten, yielding a range of 0 to 100.

The persistence dimension (denoted CPG persistence) indicated the number of days the participants were disabled by pain during the previous 4 weeks (i.e. number of days unable to work, study, or perform household work due to pain). To use the five grades of CPG, the number of days was scaled as follows: 0 point = 0–1 day; 1 point = 2–3 days; 2 points = 4–5 days; and 3 points > 5 days.

The interference dimension (CPG disability) consisted of two items: (1) how often has pain kept you from working, studying, or performing household work the previous 4 weeks and (2) how often pain has kept you from leisure activities, social activities, or family activities the previous 4 weeks. Both these items were assessed using the NRS (0 = no interference and 10 = unable to carry on these activities). CPG disability was calculated as the average of the two items and multiplying this number by ten to yield a range of 0 to 100. For the grades of CPG, the scores were converted to points according to instrument instructions: [29] 0 point = 0–29; 1 point = 30–49; 2 points = 50–69; and 3 points > 70 points. Disability points were calculated as the sum of the points for both CPG persistence and CPG disability, resulting a possible range of 0 to 6.

Next, we categorized the different grades of CPG as previously proposed: [29] (1) CPG-0: no pain and no disability (i.e. individuals without CP); (2) CPG-I: low CGP intensity (< 50) and low disability points (< 3 points); (3) CPG-II: High CGP intensity (> 50) and low disability (< 3 points); (4) CPG-III: High disability (moderately limiting; 3–4 disability points regardless of CPG intensity); and (5) CPG-IV: High disability (severely limiting; 5–6 disability points regardless of CPG intensity. The higher grade, the more pain severity. The grades of CPG were treated as an ordinal outcome to predict the cumulative ordered log-odds of being in a higher pain severity grade with reference to both intensity and disability (CPG-0, CPG-I, CPG-II, CPG-III, and CPG-IV).

### Predictor variables

Two indicators of psychological well-being within the hedonic perspective were considered—positive well-being and life satisfaction.

#### Psychological well-being

To measure well-being, we used the General Well-Being Scale (GWBS) [36]. The GWBS, a common instrument for assessing positive well-being and distress, consists of 18 items that yield a total score ranging from 0 to 110 (high score indicating positive well-being and low score indicating distress). The interval 0–60 reflects severe distress, 61–72 moderate distress, and 73–110 positive well-being. The first 14 questions use a six-point rating scale (ranging from 0 to 5) that represents intensity or frequency, and the remaining four items use an 11-point rating scale with the end-points 0 (very concerned) and 10 (not concerned at all). The instrument has provided good internal consistency, test–retest reliability, and validity. [36] GWBS can also produce six subscales: [37] Anxiety, Depression, Positive well-being, Self-control, Vitality, and General health. The data were categorized as severe distress, moderate distress, and positive well-being according to proposed intervals [36].

#### Life satisfaction

To capture the individual’s estimations of global satisfaction with life, we used the domain ‘global satisfaction’—one domain of the 11 domains listed in the Life Satisfaction Questionnaire (LISAT-11) [38]. The LISAT-11 measures levels of satisfaction along a six-point rating scale from 1 (very dissatisfied) to 6 (very satisfied). Although the LISAT-11 includes domain-specific satisfaction in ten other domains, these domains were out of the scope of our project, so we did not assess them. The data were dichotomized as ‘satisfied’ (answer options 5 and 6) and ‘not satisfied’ (answer options 1–4), as previously proposed [39]. The instrument has been shown to provide good validity [38, 39].

#### Potential confounders

We identified nine covariates (other than baseline CPGs) as potential confounders based on acknowledged associations between these factors and both CP and psychological well-being/life satisfaction: age (older adults vs. adults), sex (women vs. men), county of birth (abroad vs. born in Sweden), marital status (married vs. other), education (university vs. other), employment (unemployment vs. employment), household income (≥ median = high income vs. < median = low income), multimorbidity (multimorbidity vs. single morbidity), sleep problems (yes vs. no). Multimorbidity was assessed by a self-reported questionnaire of 12 common diagnoses of various disorders, as described elsewhere [40, 41]. Briefly, these diagnoses included traumatic injuries, rheumatoid arthritis and osteoarthritis, cardiovascular disorders, pulmonary disorders, depressive disorders, anxiety disorders, gastrointestinal disorders, disorders of the central nervous system, urogenital disorders, skin disorders, tumours and cancer, and metabolic disorders. Multimorbidity was defined as the presence of two or more diagnoses of various disorders while single morbidity was defined as the presence of one or none [42]. The assessment of sleep problems was based on a single question: Do you have trouble falling or staying asleep (Yes or No)?

### Data analysis

All statistical analyses were performed using IBM SPSS Statistics (version 23.0; IBM Inc., New York, USA). Two-sided statistical tests were used and *p* < 0.05 was regarded as significant. All analyses were stratified by the two sub-cohorts—participants without CP at T0 (CPG = 0) and participants with CP at T0 (CPG > 0). Distributions and descriptive statistics were examined for all variables at both T0 and T1. Means and standard deviations (SDs) for continuous variables and frequencies with percentages (*n*; %) for categorical variables were calculated. The associations of the baseline predictor variables (positive well-being and life satisfaction) with the outcome variable (pain severity as derived by the five ordered CPGs) were analysed using ordinal regression under a Generalized Linear Model (GLZ), which allows the outcome variable to have an ordinal distribution [43, 44]. GLZ is a flexible generalization of ordinary linear regression and can be used to analyse data with binary, discrete, or continuous outcomes [44].

We used GLZ with an ordinal distribution and a cumulative logit link function. The statistical significance of the model was examined by the Wald test. [44] Multicollinearity among covariates was estimated through variance inflation factor (VIF) with a cut-off score of > 2 as an indicator of multicollinearity [45]. Because the VIF was < 2, all covariates were included in the analysis. We present three models: one unadjusted in which crude odds ratios (ORs) with corresponding 95% confidence intervals (CIs) were calculated for both baseline predictors studied (i.e. well-being and life satisfaction) in a simultaneous model; one adjusted in which both baseline predictors studied were adjusted for the nine covariates, as previously described, excluding the baseline CPGs; and one fully adjusted model including CPGs at baseline in which both baseline predictors studied were adjusted for the nine covariates and the baseline CPGs as well. However, the fully adjusted model cannot be applied to sub-cohort 1 since everyone had a CPG = 0.

Finally, when significant associations between the baseline covariates and pain severity as measured by CPGs were found, a multiple post hoc sensitivity analysis was additionally performed to show how the interaction between well-being and life satisfaction depended on these selected participant characteristics (age, sex, county of birth, marital status, education, employment, household income, multimorbidity, and sleep problems in the association with pain severity, i.e. weaker vs. stronger).

## Results

### Study participation

The flowchart of the sample selection is depicted in Fig. 1. At T0, 15,563 individuals (46% men, 54% women) completed and returned the questionnaire, a 46% response. Of these, 11,386 (74%) completed the survey at T1. The dropout analysis showed that the response rate at T0 was lower among men, singles, and participants born abroad, and the response rate at T1 declined for younger ages, men, singles, secondary educated, unemployed, having lower household income, multimorbidity, higher functional impairment, being severely distressed, and born abroad (Table 1). Of the 11,386 individuals who participated at both T0 and T1, 2025 were further excluded because of missing data on the CPG questionnaires at either T0 or T1. Hence, 9361 individuals were included in this longitudinal study. The sub-cohort 1 and the sub-cohort 2 consisted of 5693 and 3668 participants, respectively (Fig. 1).

**Table 1:** Description of the sociodemographic characteristics and study measures at both baseline (T0) and at the 2-year follow-up (T1), and characteristics of non-participants at T0 and T1

Characteristic; *n* (%), unless otherwise stated	Number of answers on specific items	Participants at T0 (*N* = 15,563)	Number of answers on specific items	Participants at both T0 and T1 (T0 responses; *N* = 11,386)	Number of answers on specific items	Participants at both T0 and T1 (T1 responses; *N* = 11,386)	Non-Participants at T0 (*N* = 18,437)	Non-Participants at T1 (*N* = 4177)
Age, years; mean (SD)	15,563	51.6 (18.5)	11,386	53.8 (17.5)	11,384	55.8 (17.5)	–	45.6 (19.5)
Sex	15,563		11,386		11,386			
Men		7151 (46.0)		5125 (45.0)		5125 (45.0)	9837 (54.0)	2026 (48.5)
Women		8412 (54.0)		6261 (55.0)		6261 (55.0)	8382 (46.0)	2151 (51.5)
Civil status	15,555		11,381		11,386			
Single		5134 (33.0)		3283 (28.8)		3179 (27.9)	9440 (51.8)	1851 (44.3)
Married		7825 (50.3)		6104 (53.7)		6105 (53.6)	6347 (34.8)	1721 (41.2)
Divorced		1762 (11.3)		1351 (11.9)		1387 (12.2)	1802 (9.9)	411 (9.8)
Widowed		834 (5.4)		643 (5.6)		715 (6.3)	630 (3.5)	191 (4.6)
Educational level	15,256		11,205		11,162			
Elementary school		3442 (22.6)		2571 (22.9)		2491 (22.3)	–	871 (21.5)
Secondary school or vocational training		6225 (40.8)		4327 (38.6)		4257 (38.2)	–	1898 (46.9)
College or university		5589 (36.6)		4307 (38.5)		4414 (39.5)	–	1282 (31.6)
Employment status	15,115		11,102		11,002			
Employment		8708 (57.6)		6366 (57.3)		6213 (56.5)	–	2342 (58.4)
Unemployment		6407 (42.4)		4736 (42.7)		4789 (43.5)	–	1671 (51.6)
Country of birth	15,563		11,386		11,386			
Sweden		14,093 (90.6)		10,496 (92.2)		10,496 (92.2)	14,475 (79.5)	3597 (86.1)
Abroad		1470 (9.4)		890 (7.8)		890 (7.8)	3744 (20.5)	580 (13.9)
Household income, euros per year; mean (SD)	15,510	55,270 (35,157)	11,360	56,524 (34,755)	11,386	58,419 (35,919)	–	51,834 (36,014)
Co-morbidity	15,305		11,226		10,030			
Multimorbidity		4868 (31.8)		3640 (32.4)		3189 (31.8)	–	2851 (69.9)
Single morbidity		10,437 (68.2)		7586 (67.6)		6841 (68.2)	–	1228 (30.1)
Sleep problems	15,162		11,126		11,034			
Yes		5215 (34.4)		3843 (34.5)		3708 (33.6)	–	1372 (34.0)
No		9947 (65.6)		7283 (65.5)		7326 (66.4)	–	2664 (66.0)
Chronic pain grades (CPGs)	13,975		10,410		9361			
CPG-0		8234 (58.9)		6162 (59.0)		5409 (57.8)	–	2172 (58.4)
CPG-I		2414 (17.3)		1887 (18.0)		1755 (18.7)	–	584 (15.7)
CPG-II		1989 (14.2)		1463 (14.4)		1380 (14.7)	–	526 (14.1)
CPG-III		560 (4.0)		371 (3.6)		337 (3.6)	–	189 (5.1)
CPG-IV		778 (5.6)		527 (5.0)		480 (5.1)	–	251 (6.7)
Psychological well-being	14,492		10,676		10,562			
Severe distress		2769 (19.1)		1846 (17.3)		1904 (18.0)	–	923 (24.2)
Moderate distress		2556 (17.6)		1844 (17.3)		1906 (18.1)	–	712 (18.7)
Positive well-being		9167 (63.3)		6986 (65.4)		6752 (63.9)	–	2181 (57.1)
Life satisfaction	15,115		11,123		11,051			
Not satisfied		4212 (27.9)		2842 (25.5)		3005 (27.2)	–	1372 (34.0)
Satisfied		10,903 (72.1)		8281 (25.5)		8046 (72.8)	–	2664 (66.0)

*SD* standard deviation, *CPGs* chronic pain grades

### Descriptive characteristics

The total sample consisted of 4266 men (45.6%) and 5095 women (54.4%) and the mean age was 52.5 (SD = 17.5) years. In sub-cohort 1, the mean age was 51.0 (SD = 18.3) years and 49.6% were women. In sub-cohort 2, the mean age was 54.7 years (SD = 8) years and 62.0% were women. In both sub-cohorts, women were younger than men. Table 2 illustrates the baseline characteristics for the two sub-cohorts stratified by the three nominal categories of the well-being (severe distress, moderate distress, and positive well-being) and by the two nominal categories of the life satisfaction (satisfied and not satisfied).

**Table 2:** Characteristics of the two study sub-cohorts classified according to baseline psychological well-being and life satisfaction

Characteristics, *n* (%), unless otherwise stated	Psychological well-being at T0			Life satisfaction at T0	
	Severe distress (*n* = 526)	Moderate distress (*n* = 692)	Positive well-being (*n* = 4152)	Not satisfied (*n* = 1259)	Satisfied (*n* = 4377)
Study sub-cohort 1: Participants without chronic pain at T0 (CPG = 0; N1 = 5693)					
Age, mean (SD)	42.8 (17.5)	46.1 (18.7)	52.5 (17.8)	48.9 (18.9)	51.5 (17.9)
Women	308 (58.6)	395 (57.1)	1968 (47.4)	622 (49.4)	2178 (49.8)
Born abroad	43 (8.2)	63 (9.1)	235 (5.7)	111 (8.8)	249 (5.7)
Married	193 (36.7)	305 (44.1)	2299 (55.4)	485 (38.5)	2467 (56.4)
University	247 (47.9)	334 (49.0)	1810 (44.1)	493 (40.0)	2004 (46.3)
Unemployment	188 (36.6)	251 (37.1)	1592 (38.9)	513 (41.6)	1632 (37.8)
High household income (≥ median)	204 (38.8)	310 (44.8)	2286 (55.1)	550 (43.7)	2389 (54.6)
Multimorbidity	162 (34.5)	121 (19.6)	609 (15.8)	310 (28.1)	623 (15.4)
Sleep problems	272 (52.3)	255 (37.4)	748 (18.2)	485 (39.0)	837 (19.4)

Characteristics, n (%), unless otherwise stated	Psychological well-being at T0			Life satisfaction at T0	
	Severe distress (n = 923)	Moderate distress (n = 782)	Positive well-being (n = 1791)	Not Satisfied (n = 1422)	Satisfied (n = 2201)
Study sub-cohort 2: Participants with chronic pain at T0 (CPG > 0; N2 = 3668)					
Age, mean (SD)	49.9 (16.6)	53.7 (16.5)	57.1 (14.9)	54.8 (16.02)	54.5 (15.9)
Women	640 (69.3)	482 (61.6)	1049 (58.6)	888 (62.4)	1360 (61.8)
Born Abroad	111 (12.0)	76 (9.7)	87 (4.9)	151 (10.6)	129 (5.9)
Married	408 (44.3)	421 (53.9)	1064 (59.4)	691 (48.7)	1282 (52.2)
University	306 (33.6)	270 (34.7)	612 (34.3)	432 (30.7)	792 (36.3)
Unemployment	386 (42.3)	305 (39.6)	717 (40.7)	667 (47.6)	807 (37.3)
High household income (≥ median)	375 (40.6)	352 (45.0)	946 (52.8)	573 (40.3)	1154 (52.4)
Multimorbidity	458 (57.0)	296 (43.4)	606 (37.3)	639 (52.2)	770 (38.7)
Sleep problems	618 (67.5)	398 (51.9)	612 (34.5)	817 (58.3)	869 (39.9)

*SD* standard deviation, T0 baseline, *CPGs* chronic pain grades, *N* = the total number of participants according to their pain status at baseline, *n* = the total number of participants by classification according to three nominal categories of the General Well-being Schedule and to two nominal categories of the life satisfaction

### Psychological well-being and life satisfaction in relation to chronic pain grades

Percentages of well-being, life satisfaction, and CPGs were fairly stable over time for the two sub-cohorts (see Additional files 2 and 3). In sub-cohort 1 without CP at baseline, the overall proportions of positive well-being and life satisfaction were highest among CPG-0 at T1 (see Additional file 2). In sub-cohort 2 with CP at baseline, the report of severe distress and not satisfied was more noticeable for those with CPG I and II at both time points (see Additional file 3).

### Prospective associations of psychological well-being and life satisfaction in relation to chronic pain grades

The unadjusted and adjusted ordinal regression analyses for both sub-cohorts are presented in Table 3. In sub-cohort 1, the adjusted analysis (i.e. corrected for the nine baseline potential confounders) showed that participants with positive well-being had significantly lower cumulative ordered odds of being in a higher CPG grade. Life satisfaction, conversely, was not statistically associated with CPGs in sub-cohort 1 (Table 3). Furthermore, in the adjusted analysis, we found that being a woman, multimorbidity, and sleep problems were significantly associated with CPGs at T1 (see Additional file 4; Sub-cohort 1). The sensitivity analyses revealed that the association between positive well-being and pain severity is moderated by being a woman (*p* = 0.029), multimorbidity (*p* < 0.001), and sleep problems (*p* = 0.004).

**Table 3:** Ordinal regression analyses via GLZ models of CPGs at T1 using psychological well-being and life satisfaction at T0 as independent variables for the two study sub-cohorts

Baseline variables	Unadjusted			Adjusted			Fully adjusted^a^		
	OR	95% CI	*p* value	OR	95% CI	*p* value	OR	95% CI	*p* value
Sub-cohort 1: Participants without chronic pain at T0 (CPG = 0; N1 = 5693)									
Psychological well-being									
Severe distress	1 [reference]			1 [reference]					
Moderate distress	0.98	0.76–1.27	0.877	1.12	0.84–1.48	0.453	–	–	–
Positive well-being	0.53	0.42–0.67	*< 0.001*	0.64	0.49–0.84	*< 0.001*	–	–	–
Life satisfaction									
Not satisfied	1 [reference]			1 [reference]					
Satisfied	1.01	0.84–1.21	0.925	1.02	0.83–1.24	0.873	–	–	–

Baseline variables	Unadjusted			Adjusted			Fully adjusted^a^		
	OR	95% CI	*p* value	OR	95% CI	*p* value	OR	95% CI	*p* value
Sub-cohort 2: Participants with chronic pain at T0 (CPG > 0; N2 = 3668)									
Psychological well-being									
Severe distress	1 [reference]			1 [reference]			1 [reference]		
Moderate distress	0.71	0.59–0.85	*< 0.001*	0.78	0.64–0.96	*0.016*	0.96	0.78–1.17	0.654
Positive well-being	0.43	0.36–0.51	*< 0.001*	0.53	0.44–0.65	*< 0.001*	0.80	0.65-0.98	*0.031*
Life satisfaction									
Not satisfied	1 [reference]			1 [reference]			1 [reference]		
Satisfied	0.84	0.72–0.97	*0.016*	0.89	0.76–1.05	0.155	0.82	0.69–0.96	*0.014*

Italic values indicate significance of a *p* value (*p* < 0.05)

*GLZ* Generalized Linear Models for ordinal outcomes, T0 baseline, T1 2-year follow-up, *CPGs* chronic pain grades, *OR* odds ratio, *CI* Wald confidence interval, *Unadjusted* both baseline predictors were simultaneously controlled for, *Adjusted* corrected for baseline covariates excluding baseline CPGs: age (older adults vs. adults), sex (women vs. men), county of birth (abroad vs. born in Sweden), civil status (married vs. other), employment (unemployment vs. employment), education (university vs. other), household income (> median = high vs. < median = low), multimorbidity (multimorbidity vs. single morbidity), and sleep problems (yes vs. no), *Fully adjusted* corrected for all adjusted baseline covariates and baseline CPGs from I to IV

^a^The fully adjusted model cannot be applied in the sub-cohort 1 because the baseline CPG = 0

In sub-cohort 2, the adjusted analysis (i.e. corrected for the nine baseline potential confounders) showed that participants with positive well-being and moderate distress, with severe distress as a reference, had significantly lower cumulative ordered odds of being in a higher CPG grade. In this model, life satisfaction was non-significant (Table 3). The fully adjusted model including CPGs at baseline showed that participants with both positive well-being and life satisfaction had significantly lower cumulative ordered odds of being in a higher CPG grade (Table 3). Moreover, in the fully adjusted mode, we found that CPGs at T0 were associated with changes in CPGs at T1. The strongest association was found for CPG-IV compared to CPG-I (see Additional file 5).

In the fully adjusted model, we also found that being a woman, born abroad, married, university educated, multimorbidity, and sleep problems were significantly associated with CPGs at T1 (see Additional file 4; Sub-cohort 2). The sensitivity analyses revealed that the association between positive well-being and pain severity is moderated by multimorbidity (*p* < 0.040) and sleep problems (*p* = 0.016). The same analysis between life satisfaction and the above-mentioned covariates showed that none emerged as a moderator of our findings (*p* = 0.059) for the interaction for all covariates.

## Discussion

In this longitudinal study, we found that well-being at baseline, especially positive well-being, predicted lower risk of severe pain at the 2-year follow-up both for participants with no CP at baseline (sub-cohort 1) and for participants with CP (sub-cohort 2) after baseline adjustments. Although the groups with high/low well-being were quite different at baseline with respect to other background factors, the predictive associations remained after adjustments. In addition, life satisfaction was protective against increased pain severity during the same period for sub-cohort 2, but not for sub-cohort 1. Pain severity at baseline was a relatively strong significant predictor of pain severity at the 2-year follow-up. However, this adjustment did not alter the protective significant associations with positive well-being, life satisfaction, and pain severity, further enhancing confidence in our findings. Therefore, our study found that positive well-being has a favourable effect on pain severity in both sub-cohorts.

However, our findings are congruent with previous findings, which have shown that well-being is a strong predictor of positive health outcomes [3, 4, 18–20]. With respect to pain populations, our results are in agreement with findings from cross-sectional studies [23, 24]. Likewise, two prospective studies have also reported similar results [13, 14]. Briefly, in a 2-year cohort study of 1084 Mexican-Americans with arthritis, participants with high positive well-being reported a significantly lower risk of disability (almost 55%) after controlling for various baseline cofounders [14]. A considerable related result emerged from another longitudinal study including a total of 13,594 participants aged ≥ 50 years and with a follow-up of over 9 years [13]. However, we cannot directly compare our results with other studies because of dissimilar populations, outcomes, and assessments of well-being. The possibility that a reciprocal association between well-being and ill-being may exist should also be considered [1, 46]. In any case, further prospective epidemiological research is required to confirm the temporal links between illness and well-being in pain populations.

Our results suggest a more protective effect of well-being in sub-cohort 1 compared to sub-cohort 2. This effect can be attributed to the fact that diseased populations, especially those in chronicity, have lower cumulative exposure to well-being than healthy populations and greater exposure to ill-being over time. Nevertheless, the difference in ORs was not very large between the two sub-cohorts, indicating an overall favourable effect of well-being in general populations with and without pain. The finding that life satisfaction was associated with less pain severity in sub-cohort 2 but not in sub-cohort 1, although not easy to interpret, may indicate that positive evaluations of life may vary across a lifespan depending on the context they occurred [47]. Thus, there is the possibility that diseased populations have a better hedonic adaptation—i.e. these populations evaluate their personal success and way of living more positively and as such this belief serves as a defence mechanism against illness, whereas in healthy populations life satisfaction may decline over time [48]. These speculations require further examination.

Testing a model containing baseline pain adjustments in sub-cohort 2, we found that participants who reported high severe pain at baseline had more than ten times increased risk of severe pain at the 2-year follow-up. These results provide further evidence that pain severity is the strongest predictor of worse future pain severity. More importantly, these results demonstrate that well-being and pain severity are independently and differentially associated with pain severity risk, at least in pain populations. Additionally, the association between positive well-being and reduced risk of pain severity seems to be partly conditioned by other baseline factors in both sub-cohorts. In sub-cohort 1, we found that the association was weaker in women compared to men, in multimorbidity compared to single morbidity, and in sleep problems compared to no sleep problems. In sub-cohort 2, we found that the association was weaker only in multimorbidity compared to single morbidity and in sleep problems compared to no sleep problems. Our results are consistent with previous studies that displayed a significant association between well-being, sex, and morbidities with higher well-being in men [49] and lower in patients with multimorbidities [46]. In contrast, none of the baseline covariates emerged as a moderator between the association of life satisfaction and pain severity.

### Strengths and limitations

This is the first study to reveal a predictive value of positive well-being and life satisfaction in a large cohort of the general population with and without chronic pain, alongside with its prospective nature, detailed exposure data, and confounder information. Furthermore, two internationally accepted and commonly used measures of well-being [38, 39] within the hedonic perspective were chosen to address our study hypothesis [1, 2]. However, our study has some shortcomings. One limitation is that all the assessments reported here are based on self-report measurements, so it is possible that our results are overestimated. It has been reported that self-reported health measures are more likely to exhibit biased associations because they exhibit stronger associations between well-being and health outcomes than health assessments made by physicians [50]. Although we had sufficient sample sizes per nominal category of well-being and life satisfaction, the proportion of participants reported higher well-being and life satisfaction was substantially larger. Thus, a ceiling effect might be presented [51]. Furthermore, we observed that the response rate at follow-up weakened among individuals with stronger indicators of biopsychological stress, indicating a response bias or a selection bias. Finally, we did not adjust for baseline-specific chronic pain conditions (i.e. traumatic or non-traumatic neck pain, low back pain, fibromyalgia). These adjustments should be considered in future studies.

Acknowledging these caveats, our study highlights the importance of well-being not only as an additional important factor worthy to be assessed in clinical daily practice, but also as a possible new treatment option in pain populations. Although the pathways connecting well-being to positive outcomes remain unknown, there is evidence that well-being may receive its protective effect through three mechanisms [7, 16, 52–54]. It has been proposed that it can effectively reinforce one’s physiological and biological functioning, and the effective functioning, in turn, retains the health state status in healthy populations or may benefit recovery in diseased populations [7, 16, 52–54]. It can further promote individuals to embrace healthy habits and practices such as cessation of smoking, physical exercise, weight control, and healthy sleep habits [7, 9, 10]. Lastly, some theoretical models proposed that well-being can buffer the negative effects of stress by helping individuals embrace adaptive coping skills for stressful life events [19, 55]. However, it is not known if these three mechanisms function independently, are inter-correlated, or are simply reversed associations [1]. For this reason, future experimental studies should examine more thoroughly these pathways of positive well-being in pain populations.

## Conclusions

This study found that hedonic measures of well-being are protective factors against pain severity. Well-being, therefore, should be considered when designing or modifying pain treatment interventions. This study also suggests that health care providers should examine ways of improving and preserving positive well-being in populations with and without pain. The potential pathways linking well-being to positive pain-related outcomes must also be thoroughly examined in future studies, since pain appears to have a complex biological, psychological, and social function. Future studies may want to assess overall well-being using the three perspectives (i.e. hedonic, eudaimonic, and social) to identify the independent associations related to positive pain-related outcomes.

## Additional files

**Additional file 1.** STROBE Statement.

**Additional file 2: Figure S1.** The relation between psychological well-being, life satisfaction at T0, and pain severity according to CPGs at T1 for sub-cohort 1: Participants without chronic pain at T0 (CPG = 0) Notes: CPGs = Chronic pain grades, T0 = baseline, T1 = 2-year follow-up.

**Additional file 3: Figure S2.** The relation between psychological well-being, life satisfaction at T0, and pain severity classified according to CPGs at both T0 and T1 for sub-cohort 2: Participants with chronic pain at T0 (CPGs > 0). Notes: CPGs = Chronic pain grades, T0 = baseline, T1 = 2-year follow-up.

**Additional file 4: Figure S3.** Forest plot (OR and 95% CI) summarizing the results of the ordinal regression analysis via GLZ models for the nine baseline covariates for the association between these covariates at T0 and CPGs at T1 for the sub-cohort 1: Participants without chronic pain at T0 (CPG = 0) (**left;** adjusted model) and sub-cohort 2: Participants with chronic pain at T0 (CPGs > 0) (**right;** fully adjusted model). An OR > 1 increases the odds of pain severity; an OR < 1 decreases the odds of pain severity. Notes: OR = Odds ratio, CI = Confidence interval, GLZ = Generalized Linear Models for ordinal outcomes. CPGs = Chronic pain grades, T0 = baseline, T1 = 2-year follow-up.

**Additional file 5: Figure S4.** Forest plot (OR and 95% CI) summarizing the results of the ordinal regression analysis via GLZ models for the changes of CPGs from T0 to T1 for only the sub-cohort 2: Participants with chronic pain at T0 (CPGs > 0; fully adjusted model). An OR > 1 increases the odds of pain severity; an OR < 1 decreases the odds of pain severity. Notes: OR = Odds ratio, CI = Confidence interval, GLZ = Generalized Linear Models for ordinal outcomes. CPG = Chronic pain grades, T0 = baseline, T1 = 2-year follow-up.

## Data Availability

Due to the Swedish law regarding the type of data including grounds of confidentiality and anonymity, data will not be available.

## Abbreviations

CI: confidence interval
CP: chronic pain
CPG: chronic pain grade
GLZ: Generalized Linear Model
GWBS: the General Well-Being Scale
LISAT: Life Satisfaction Questionnaire
NRS: numeric rating scale
OR: odds ratio
SD: standard deviation
STROBE: Strengthening the Reporting of Observational Studies in Epidemiology
VIF: variance inflation factor
