# Supplementary material for: Positive psychological well-being predicts lower severe pain in the general population: a 2-year follow-up study of the SwePain cohort
Source: Ann Gen Psychiatry. 2019 May 31;18:8. doi: 10.1186/s12991-019-0231-9 (PMC6543656; doi:10.1186/s12991-019-0231-9)
Supplement: Supplementary file 1 — Additional file 1. STROBE Statement. [file 12991_2019_231_MOESM1_ESM.doc]

**Additional file**

**Additional file S1** STROBE Statement

—checklist of items that should be included in reports of observational studies

|  | Item No | Recommendation | Page  #Line |
| --- | --- | --- | --- |
| **Title and abstract** | 1 | (*a*) Indicate the study’s design with a commonly used term in the title or the abstract | 1 #1-2 |
|  |  | (*b*) Provide in the abstract an informative and balanced summary of what was done and what was found | 2 # 31-53 |
| Introduction | | |  |
| Background/rationale | 2 | Explain the scientific background and rationale for the investigation being reported | 4-5 # 76-11 |
| Objectives | 3 | State specific objectives, including any prespecified hypotheses | 5 #111-118 |
| Methods | | |  |
| Study design | 4 | Present key elements of study design early in the paper | 5 #120-126 |
| Setting | 5 | Describe the setting, locations, and relevant dates, including periods of recruitment, exposure, follow-up, and data collection | 6 # 127-138 |
| Participants | 6 | (*a*) *Cohort study*—Give the eligibility criteria, and the sources and methods of selection of participants. Describe methods of follow-up  *Case-control study*—Give the eligibility criteria, and the sources and methods of case ascertainment and control selection. Give the rationale for the choice of cases and controls  *Cross-sectional study*—Give the eligibility criteria, and the sources and methods of selection of participants | 5 # 139-149 |
|  |  | (*b*) *Cohort study*—For matched studies, give matching criteria and number of exposed and unexposed  *Case-control study*—For matched studies, give matching criteria and the number of controls per case | Not applicable |
| Variables | 7 | Clearly define all outcomes, exposures, predictors, potential confounders, and effect modifiers. Give diagnostic criteria, if applicable | 7-10 #155-229 |
| Data sources/ measurement | 8* | For each variable of interest, give sources of data and details of methods of assessment (measurement). Describe comparability of assessment methods if there is more than one group | 7-10 #155-229 |
| Bias | 9 | Describe any efforts to address potential sources of bias | 10-11 # 230-261 |
| Study size | 10 | Explain how the study size was arrived at | Figure 1 |
| Quantitative variables | 11 | Explain how quantitative variables were handled in the analyses. If applicable, describe which groupings were chosen and why |  |
| Statistical methods | 12 | (*a*) Describe all statistical methods, including those used to control for confounding | 10-11 # 230-261 |
|  |  | (*b*) Describe any methods used to examine subgroups and interactions | 11 # 255-260 |
|  |  | (*c*) Explain how missing data were addressed | Figure 1 |
|  |  | (*d*) *Cohort study*—If applicable, explain how loss to follow-up was addressed  *Case-control study*—If applicable, explain how matching of cases and controls was addressed  *Cross-sectional study*—If applicable, describe analytical methods taking account of sampling strategy | Supplementary Table 1 ; Figure 1 |
|  |  | (*e*) Describe any sensitivity analyses | 11 # 255-260 |

| Results | | |  |
| --- | --- | --- | --- |
| Participants | 13 | (a) Report numbers of individuals at each stage of study—eg numbers potentially eligible, examined for eligibility, confirmed eligible, included in the study, completing follow-up, and analysed | 11 # 262-272 |
|  |  | (b) Give reasons for non-participation at each stage | 11 # 265-269 |
|  |  | (c) Consider use of a flow diagram | Supplementary Table 1 ; Figure 1 |
| Descriptive data | 14 | (a) Give characteristics of study participants (eg demographic, clinical, social) and information on exposures and potential confounders | 12 # 274-287  Table 1 |
|  |  | (b) Indicate number of participants with missing data for each variable of interest | Figure 1 |
|  |  | (c) *Cohort study*—Summarise follow-up time (eg, average and total amount) | Supplementary Table 1 ; Figures 1-3 |
| Outcome data | 15 | *Cohort study*—Report numbers of outcome events or summary measures over time | Supplementary Table 1 ; Figure 1 |
|  |  | *Case-control study—*Report numbers in each exposure category, or summary measures of exposure |  |
|  |  | *Cross-sectional study—*Report numbers of outcome events or summary measures |  |
| Main results | 16 | (*a*) Give unadjusted estimates and, if applicable, confounder-adjusted estimates and their precision (eg, 95% confidence interval). Make clear which confounders were adjusted for and why they were included | 12-13 # 290-314  Table 2 |
|  |  | (*b*) Report category boundaries when continuous variables were categorized |  |
|  |  | (*c*) If relevant, consider translating estimates of relative risk into absolute risk for a meaningful time period |  |
| Other analyses | 17 | Report other analyses done—eg analyses of subgroups and interactions, and sensitivity analyses | 12-13 # 290-314  Figures 4-5 |
| Discussion | | |  |
| Key results | 18 | Summarise key results with reference to study objectives | 23-14 # 315-327 |
| Limitations | 19 | Discuss limitations of the study, taking into account sources of potential bias or imprecision. Discuss both direction and magnitude of any potential bias | 14 # 332-334 |
| Interpretation | 20 | Give a cautious overall interpretation of results considering objectives, limitations, multiplicity of analyses, results from similar studies, and other relevant evidence | 14-17 # 345-401 |
| Generalisability | 21 | Discuss the generalisability (external validity) of the study results | 14 # 328-330 |
| Other information | | |  |
| Funding | 22 | Give the source of funding and the role of the funders for the present study and, if applicable, for the original study on which the present article is based | 17 # 413-419 |
