# Supplementary figures and images for: Positive psychological well-being predicts lower severe pain in the general population: a 2-year follow-up study of the SwePain cohort
Source: Ann Gen Psychiatry. 2019 May 31;18:8. doi: 10.1186/s12991-019-0231-9 (PMC6543656; doi:10.1186/s12991-019-0231-9)

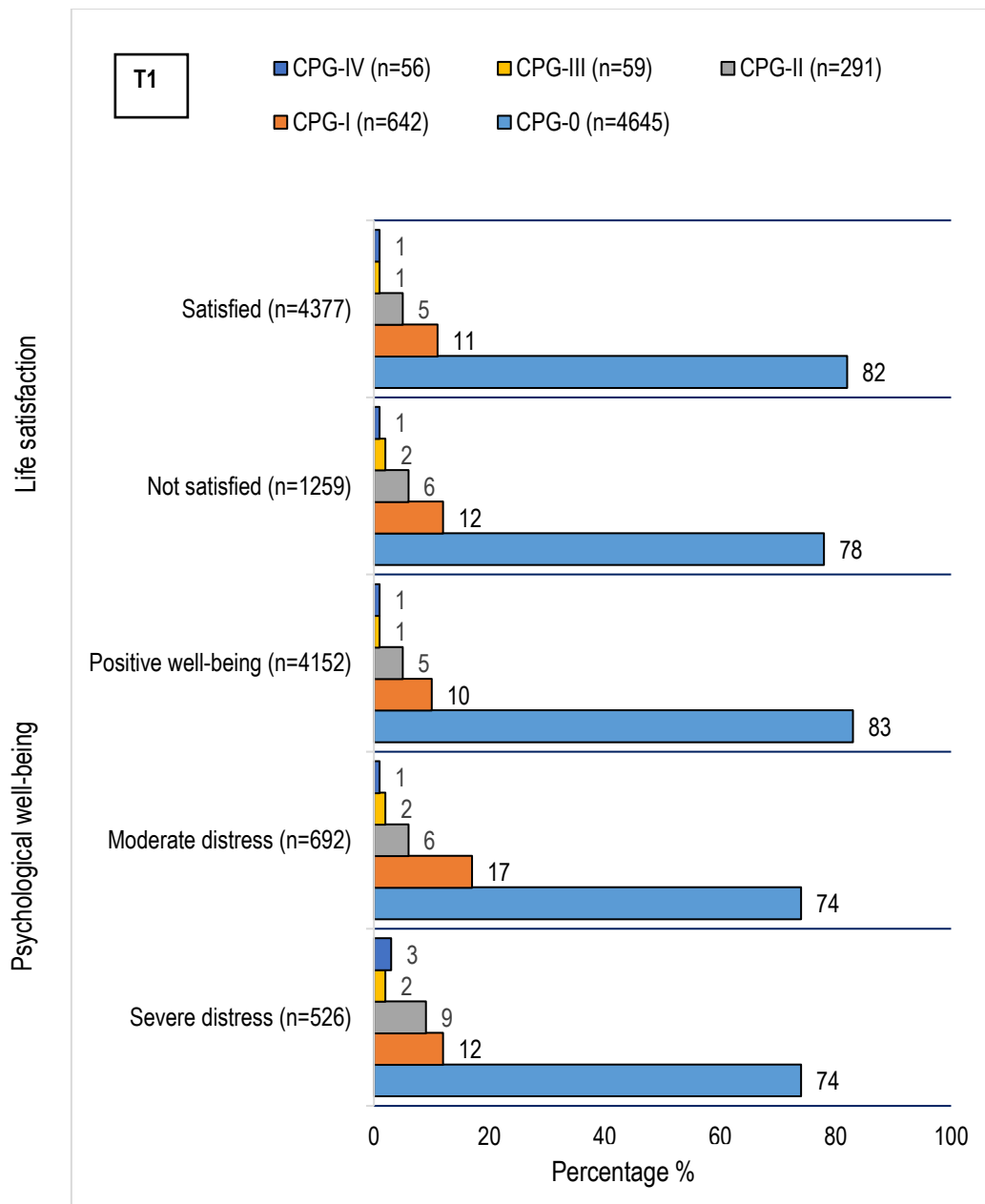

Supplement: Supplementary file 2 — Additional file 2: Figure S1. The relation between psychological well-being, life satisfaction at T0, and pain severity according to CPGs at T1 for sub-cohort 1: Participants without chronic pain at T0 (CPG = 0) Notes: CPGs = Chronic pain grades, T0 = baseline, T1 = 2-year follow-up. [file 12991_2019_231_MOESM2_ESM.pdf]

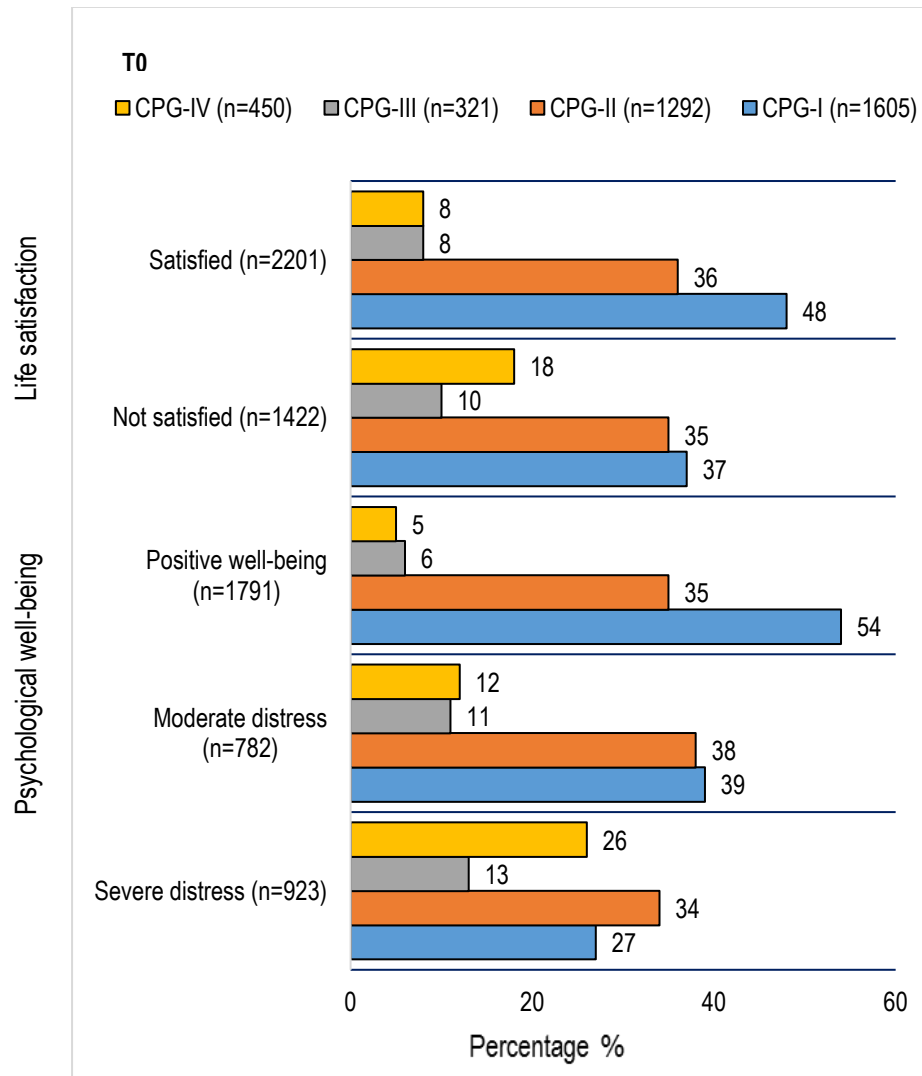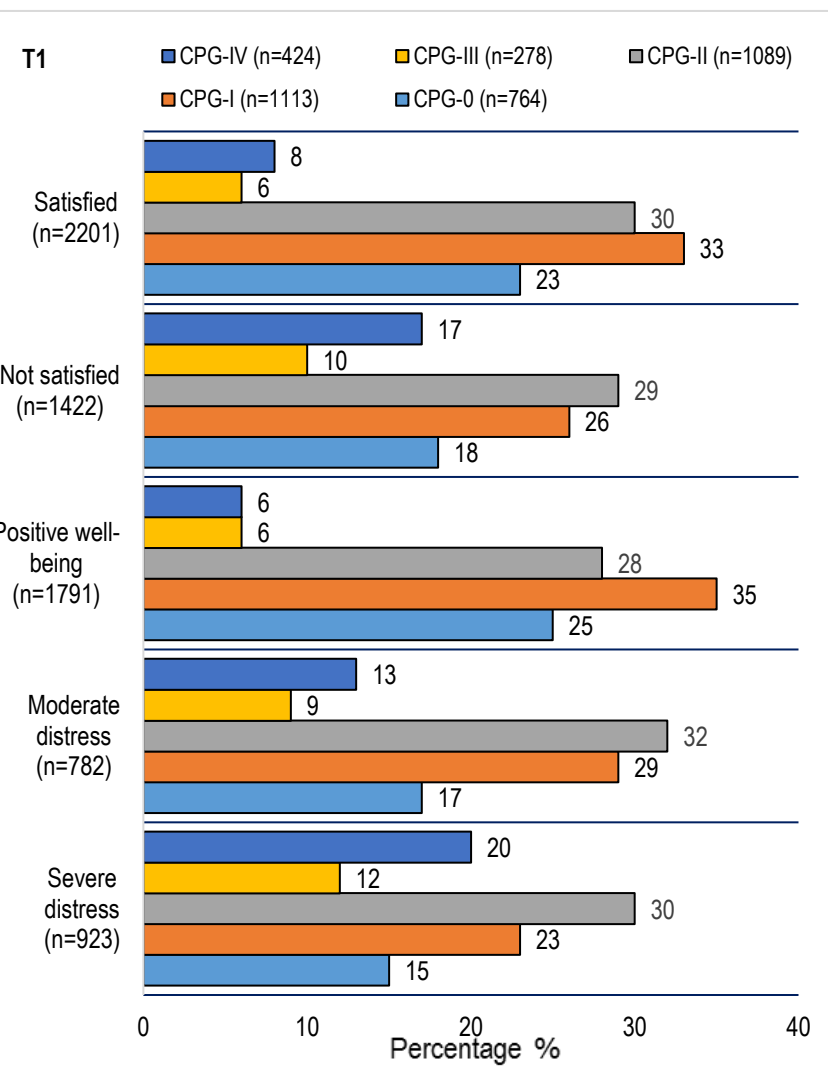

Supplement: Supplementary file 3 — Additional file 3: Figure S2. The relation between psychological well-being, life satisfaction at T0, and pain severity classified according to CPGs at both T0 and T1 for sub-cohort 2: Participants with chronic pain at T0 (CPGs > 0). Notes: CPGs = Chronic pain grades, T0 = baseline, T1 = 2-year follow-up. [file 12991_2019_231_MOESM3_ESM.pdf]

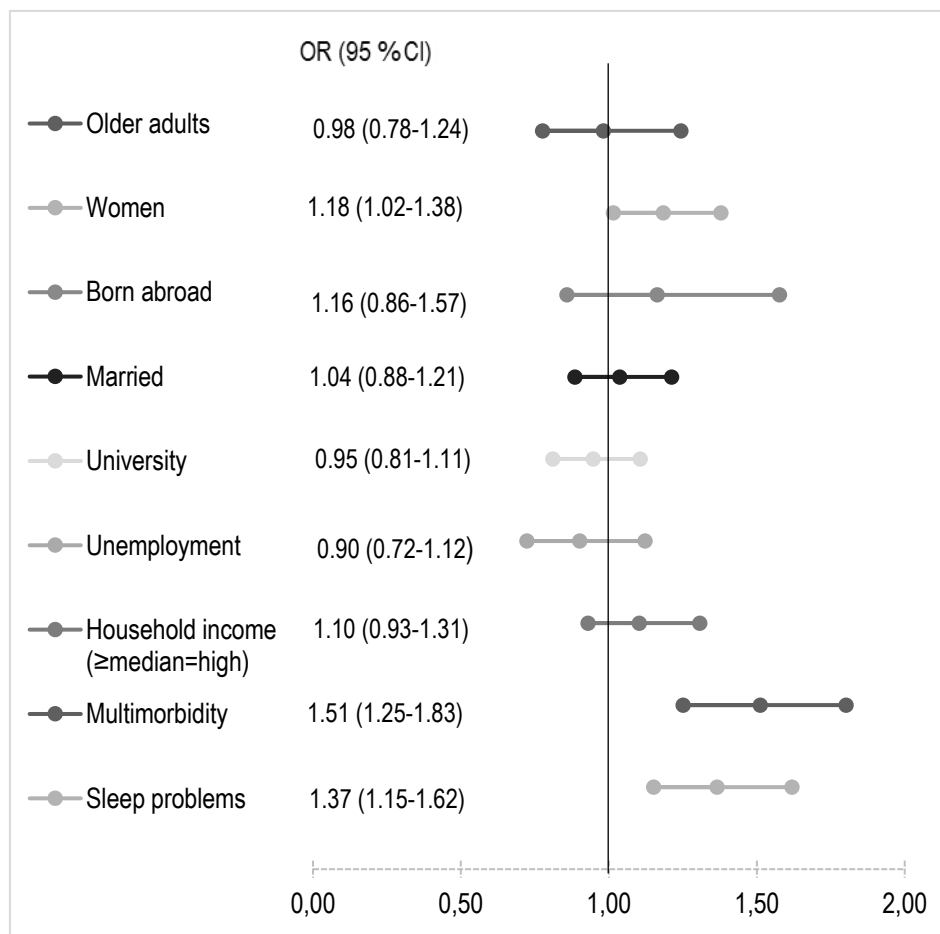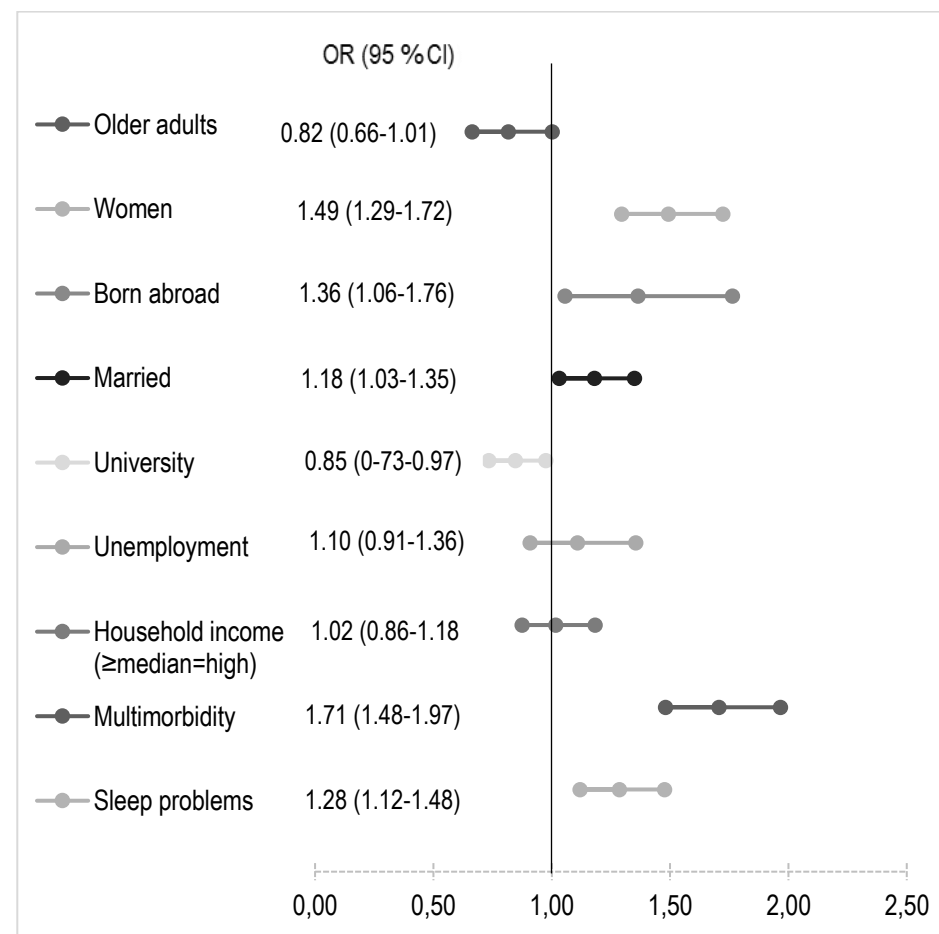

Supplement: Supplementary file 4 — Additional file 4: Figure S3. Forest plot (OR and 95% CI) summarizing the results of the ordinal regression analysis via GLZ models for the nine baseline covariates for the association between these covariates at T0 and CPGs at T1 for the sub-cohort 1: Participants without chronic pain at T0 (CPG = 0) (left; adjusted model) and sub-cohort 2: Participants with chronic pain at T0 (CPGs > 0) (right; fully adjusted model). An OR > 1 increases the odds of pain severity; an OR < 1 decreases the odds of pain severity. Notes: OR = Odds ratio, CI = Confidence interval, GLZ = Generalized Linear Models for ordinal outcomes. CPGs = Chronic pain grades, T0 = baseline, T1 = 2-year follow-up. [file 12991_2019_231_MOESM4_ESM.pdf]

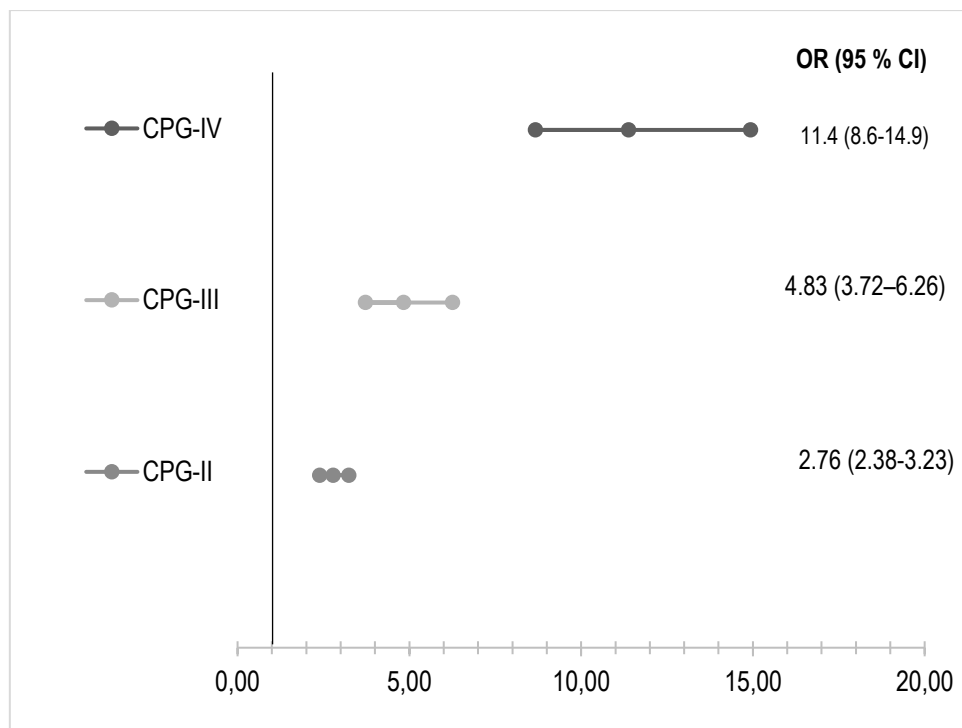

Supplement: Supplementary file 5 — Additional file 5: Figure S4. Forest plot (OR and 95% CI) summarizing the results of the ordinal regression analysis via GLZ models for the changes of CPGs from T0 to T1 for only the sub-cohort 2: Participants with chronic pain at T0 (CPGs > 0; fully adjusted model). An OR > 1 increases the odds of pain severity; an OR < 1 decreases the odds of pain severity. Notes: OR = Odds ratio, CI = Confidence interval, GLZ = Generalized Linear Models for ordinal outcomes. CPG = Chronic pain grades, T0 = baseline, T1 = 2-year follow-up. [file 12991_2019_231_MOESM5_ESM.pdf]
